# Rapid detection and differentiation of *Salmonella *species, *Salmonella* Typhimurium and *Salmonella* Enteritidis by multiplex quantitative PCR

**DOI:** 10.1371/journal.pone.0206316

**Published:** 2018-10-25

**Authors:** Raymond Heymans, Amir Vila, Caroliene A. M. van Heerwaarden, Claudia C. C. Jansen, Greetje A. A. Castelijn, Menno van der Voort, Elisabeth G. Biesta-Peters

**Affiliations:** Netherlands Food and Consumer Product Safety Authority, Directorate Enforcement, Laboratories division, Laboratory for Feed and Food Safety & Product Safety, Wageningen, the Netherlands; University of Campinas, BRAZIL

## Abstract

A multiplex quantitative PCR (qPCR) was developed and evaluated for the simultaneous detection of *Salmonella* spp., *S*. *enterica* serovar Typhimurium and *S*. *enterica* serovar Enteritidis in various (food) matrices. Early and fast detection of these pathogens facilitates effective intervention and prevents further distribution of contaminated food products on the market. Three primer and probe sets were designed to target the *invA* gene, the STM4200 gene, and the SEN1392 gene to detect and differentiate *Salmonella* spp., *S*. Typhimurium, and *S*. Enteritidis, respectively. The multiplex qPCR targeting these three genes was optimized for efficiency and linearity. By testing 225 *Salmonella* isolates and 34 non-*Salmonella* isolates from various sources the inclusivity and exclusivity were determined. The inclusivity of the multiplex qPCR was 100% for all *Salmonella* isolates, including 72 *S*. Typhimurium isolates, and 53 *S*. Enteritidis isolates. The exclusivity for *Salmonella* spp., *S*. Typhimurium, and *S*. Enteritidis was 100%, 94.6%, and 100%, respectively. No positive results were reported for non-*Salmonella* isolates. The limit of detection (LOD) for the qPCR was determined for the matrices poultry, minced meat, egg, herbs/spices, powdered milk, fish, animal feed, boot-socks with chicken feces and chicken down. LOD values for qPCR and the conventional culture methods were similar, except for the matrix boot-socks and down, for which the LOD for the conventional culture methods performed better than the qPCR method. In conclusion, the multiplex qPCR assay developed allows for rapid screening of *Salmonella* spp., *S*. Typhimurium, and *S*. Enteritidis in various (food) matrices.

## 1. Introduction

Enteric salmonellosis imposes a major burden on public health in both underdeveloped and industrialized countries. It is estimated that annually 93.8 million cases of non-typhoidal *Salmonella* gastroenteritis and 155,000 cases of mortality occur worldwide [1–3]. Many cases of non-typhoidal *S*. *enterica* infections are zoonotic since the intestinal tract of various domestic and wild animals are a natural reservoir for this pathogen [4,5]. Serious enteric illness may be caused by cross contamination of food with fecal matter of infected animals, fecal-oral transmission from infected human, or contamination from environmental or other food sources [6,7]. These diverse means of transmission emphasize the need to maintain strict hygiene regimens concerning food handling at every stage of the food production chain to prevent cross contamination and ensure food safety [8,9].

*S*. *enterica* subsp. *enterica* serovars Typhimurium and Enteritidis were the most frequently reported serovars in Europe and were responsible for the majority of cases of human salmonellosis in 2011 [6,10]. Monophasic *S*. Typhimurium 1,4,[5],12:i:- was the third most commonly reported serovar in the EU, followed by *S*. Infantis in fourth place.

Member states are now required to systematically test poultry products for the serovars *S*. *enterica* subsp. *enterica* serovars Typhimurium and Enteritidis [11]. To detect *Salmonella* in food samples in general, molecular testing in addition to conventional culture-based methods can be used. Multiplex quantitative PCR (qPCR) has proven to be a fast, easy to perform, and sensitive molecular technique for the detection of *Salmonella* species and various *Salmonella* serovars [12–17]. Screening of enrichments by qPCR will indicate within 24 hours if an enrichment is *Salmonella* negative or positive, while a culture based method is more laborious and time-consuming to perform, requiring an additional 24 hours to produce a result. Early detection of *Salmonella* positive samples by qPCR facilitates earlier intervention and prevention of further distribution of contaminated food products on the market compared to culture based methods.

In this study a *Salmonella* multiplex qPCR for the detection and differentiation of *Salmonella* spp., *S*. Typhimurium, and *S*. Enteritidis, was developed. The multiplex qPCR could be used for the primary screening of enriched samples from a variety of matrices, and for the confirmation of *Salmonella* suspected isolates. The multiplex qPCR method was validated by determining the PCR efficiency, relative accuracy and selectivity. In addition, the level of detection (LOD) of the multiplex qPCR was compared to the LOD of two culture-based *Salmonella* reference methods; being the ISO method for *Salmonella* detection [18] and the modified ISO method on Modified Semisolid Rappaport-Vassiliadis (MSRV) medium, hereafter referred to as the MSRV method [18]. The matrices tested concerned poultry, minced meat, egg, herbs/spices, powdered milk, fish, animal feed, socks with chicken feces, swabs with debris and chicken down. These matrices were chosen for two reasons. Firstly, the matrices selected are known to be relevant sources for *Salmonella* occurrence and growth [6]. Secondly, ISO 16140–2:2016 [19] specifies the general principle and the technical protocol for the validation of alternative methods for microbiology in the food chain. It recommends choosing matrices from at least five categories of food to allow the validated method to be applied for a broad range of foods. By selecting feed samples, environmental samples, and primary production stage samples, the application of this method is broadened. This suggested approach was followed in this research to obtain a validated and sensitive method which can be applied for screening for *Salmonella* spp., *S*. Enteritidis and *S*. Typhimurium in a broad range of foods, feed, environmental samples and production stage samples.

## 2. Materials and methods

### Primer and probe design

To identify serovar-specific genomic regions to differentiate *Salmonella* spp. and *S*. Typhimurium or *S*. Enteritidis from other relevant *Salmonella* serovars, whole-genome alignments were performed by using VISTA Tools [20, 21]. STM4200, a putative phage tail fiber protein of *S*. Typhimurium, and SEN1392, a predicted phage protein specific for *S*. Enteritidis, were identified as potential target genes for the differentiation of *S*. Typhimurium and *S*. Enteritidis, respectively and these genes were targeted in this study. Primers and dual-labeled hydrolysis probes were designed to target a 101 bp gene fragment of STM4200 and a 77 bp gene fragment of SEN1392 (Table 1). As *invA* is by many considered a standard for the detection of *Salmonella* spp. [22], a 95 bp product for the *invA* gene was amplified using a newly developed primer and hydrolysis probe set that was compatible with the current multiplex format.

**Table 1:** Primers and probe sets designed to target *invA* and the selected gene fragments of STM4200 and SEN1392.

*Gene*	Forward (F)/ Reverse (R)/ Probe (P)	Primer sequence (5’-3’)	PCR product size (bp)	Primer /probe concentration (nM)	Annotation	Gene reference
*invA*	F	GCTGCTTTCTCTACTTAAC	95^a^	200	Invasion protein *invA*	[17]
	R	GTAATGGAATGACGAACAT		200		
	P	FAM- CATCACCATTAGTACCAGAATCAGT-BHQ1		200		
STM4200	F	CACCTGATATAGAGTCCAA	101^a^	200	Putative phage tail fiber protein	This study
	R	TATAGATGTTGTCGCCAA		200		
	P	Cy5- AAGGTATTCTTGACTGAACAATGCC-BHQ1		200		
SEN1392	F	GGATATGAGGTGCGTTTA	77^b^	200	Predicted phage protein	[24]
	R	CAGTGCCGGAATTATCTC		200		
	P	HEX- CACCATGACCCGCAGACG-BHQ1		200		
IAC^c^	F	GATCAGCTACGTGAGGTCCTAC	145	200		[25]
	R	CTAACCTTCGTGATGAGCAATCG		200		
	P	TEXAS-RED-AGCTAGTCGATGCACTCCAGTCCTCCT		150		

^a^*S*. Typhimurium LT2

^b^*S*. Enteritidis P125109

^c^IAC primers

To check the specificity of the selected primers, the primers were tested by BlastN [23]. An NCBI Entrez dataset with a known number of entries of completed genomes (excluding contig entries) was created to test the inclusivity. The dataset contained 445 *Salmonella* species entries, including 204 *S*. Enteritidis and 49 *S*. Typhimurium entries. To test the exclusivity, primers were also subjected to a general BlastN search against the Nucleotide collection. All BlastN searches were performed July 21, 2017. Blast hits with an identity score lower than 80% were regarded as negative, when taking the full length of the oligo’s into consideration (100% coverage).

### Multiplex qPCR assay development

To avoid competition in coamplifying multiple targets and to minimize reaction volumes to reduce costs, the qPCR conditions were optimized. An internal amplification control (IAC) was included in the multiplex qPCR to detect PCR inhibition and in that way to exclude false negative qPCR reactions [12,25]. For singleplex PCRs the PCR products of the reference strains were analyzed by capillary electrophoresis using a 2100 Bioanalyzer (Agilent, USA) to investigate if one single DNA fragment was produced and to determine the size of the fragments. A one Kb DNA Ladder (Agilent, United States) was used as a molecular weight standard.

The development of the Multiplex qPCR assay was followed by determining the performance characteristics efficiency, accuracy, selectivity, and LOD.

### Efficiency

To determine the qPCR efficiency, standard curves were constructed for *S*. Typhimurium WDCM 00031, and *S*. Enteritidis WDCM 00119. The strains were taken from the -80°C freezer, one bead from each cryotube was streaked onto Tryptic Soy Agar (TSA; Biotrading, Mijdrecht, The Netherlands) and the TSA plate was incubated overnight for 24 ± 2 h at 37 ± 1°C. Five colonies per plate were selected and suspended in reversed osmoses (RO) water in Eppendorf tubes at a concentration of 3.5 McFarland (~10^9^ CFU/ml). DNA was extracted from the suspension by heating 10 min at 95°C (Thermomixer compact, Eppendorf, Hamburg, Germany) at 800 RPM, followed by centrifugation for 5 min at 10,000 RPM (Eppendorf centrifuge 5415, Eppendorf, Germany). A serial dilution was made from the DNA extract in triplicate and each dilution was analyzed in duplicate using the qPCR method developed in this study.

### Relative accuracy

To determine the correlation between the C_q_-value and the log CFU present in a sample, cell standard curves were constructed for *S*. Typhimurium WDCM 00031, and *S*. Enteritidis WDCM 00119 in Buffered Peptone Water (BPW; Biotrading, Mijdrecht, The Netherlands), with and without the presence of a food matrix (poultry and curcuma spice). The strains were taken from the -80°C freezer, one bead from each cryotube was streaked onto TSA, and incubated overnight for 24 ± 2h at 37 ± 1°C. Five colonies per plate were selected from the incubated plates and suspended in BPW in Eppendorf tubes at a concentration of 3.5 McFarland (~10^9^ CFU/ml). From here serial dilutions were made in BPW, BPW with curcuma spice and BPW with poultry. Serial dilutions for each matrix were made in triplicate. The contamination level (CFU/ml) of the dilutions was determined by plating the dilutions onto TSA, incubating the plates 24 ± 2h at 37 ± 1°C and subsequent enumeration. DNA was extracted from the dilution series using the KingFisher Flex Purification System (Thermo Fisher Scientific, Breda, The Netherlands) using the QuickPick Plant DNA kit (Bio-Nobile, Pargas, Finland) according to manufacturer’s instructions. Every DNA extract was analyzed in duplicate using the qPCR method developed in this study.

### Selectivity

A total of 225 *Salmonella* isolates (Table 2) were selected and amplified with the *Salmonella* multiplex qPCR for selectivity testing, i.e. determining the absence or presence of the targeted genes *invA*, STM4200 and SEN1392. The *invA* gene was expected to be detected in all *Salmonella* strains, whereas the STM4200 and SEN1392 genes were only expected to be detected in *S*. Typhimurium and *S*. Enteritidis strains respectively. Thirteen of these *Salmonella* isolates were reference strains obtained from the National Collection of Type Cultures (NCTC, Public Health England, Salisbury, UK). The remaining 212 non-commercial *Salmonella* isolates were obtained from various food and animal sources from the collection of the Laboratory for Feed and Food Safety & Product Safety of the Netherlands Food and Consumer Product Safety Authority, Ministry of Agriculture, Nature and Food Quality, Wageningen, the Netherlands. In addition, 34 non-*Salmonella* reference strains were included for selectivity testing (Table 3).

**Table 2:** Detection of the *invA*, STM4200 and SEN1392 genes for 225 *Salmonella* strains by the developed multiplex qPCR methods, where (+) indicates detection of the gene and (-) indicates the gene is not detected.

Strain		Multiplex real-time PCR results			
***S*. Typhimurium**	**n**	***invA***	**STM4200**	**SEN1392**	**Source of sample(s)**
*S*. Typhimurium	62	+	+	-	Duck meat, turkey meat, chicken meat (n = 9), chicken eggs (n = 4), fowl organ meat, chicken carcass, sheep meat, horse meat, bovine (minced) meat (n = 7), bovine feces (n = 3), pork (minced) meat (n = 11), (minced) meat of unknown source (n = 9), sausage (n = 9), swab, chicken soup, baby food, protein powder
*S*. Typhimurium monophasic 4,[5],12,i;-	5	+	+	-	Swab from swine, pork sausage, flavorings, bovine feces (n = 2)
*S*. Typhimurium monophasic 4,12,i;-	2	+	+	-	Pork sausage, pork
*S*. Typhimurium WDCM 00031	1	+	+	-	Reference Strain
*S*. Typhimurium WDCM 00121	1	+	+	-	Reference Strain
*S*. Typhimurium WDCM 00122	1	+	+	-	Reference Strain
***S*. Enteritidis**	**n**	***invA***	**STM4200**	**SEN1392**	**Source of sample(s)**
*S*. Enteritidis	52	+	-	+	Chicken meat (n = 13), chicken eggs (n = 11), fowl organ meat, sheep swab, bovine (minced) meat (n = 3), pork meat (n = 6), shellfish (n = 3), sausage (n = 2), powdered milk, protein powder (n = 5), flour, powdered sweet pepper, pet food (n = 3), human feces
*S*. Enteritidis WDCM 00119	1	+	-	+	Reference Strain
***Salmonella* Serotypes**	**n**	***invA***	**STM4200**	**SEN1392**	**Source of sample(s)**
*S*. Agona	3	+	-	-	Chicken meat, bovine feces, cumin seed
*S*. Anatum	2	+	-	-	Chicken carcasses, powdered milk
*S*. Bareilly	1	+	-	-	Powdered milk
*S*. Blockley	1	+	-	-	Chicken meat
*S*. Bovis-morbificans	1	+	-	-	Minced meat
*S*. Braenderup	1	+	-	-	Protein powder
*S*. Brandenburg	1	+	-	-	Sausage
*S*. Bredeney	1	+	-	-	Sausage
*S*. Cerro	1	+	-	-	Protein
*S*. Derby	2	+	+	-	Chicken meat, pork
*S*. Dublin	7	+	-	-	Bovine feces (n = 2), bovine (minced) meat (n = 4), bovine sausage,
*S*. Gallinarum NCTC 10532	1	+	-	-	Reference strain
*S*. Give	1	+	-	-	Mixed meats (hare, hog,deer)
*S*. Goldcoast	1	+	+	-	Sausage
*S*. Hadar	5	+	-	-	Chicken carcasses (n = 2), chicken meat (n = 2), basil,
*S*. Heidelberg	5	+	-	-	Chicken meat (n = 5)
*S*. Indiana	2	+	-	-	Chicken carcasses, fowl organ meat
*S*. Infantis	5	+	-	-	Chicken carcasses, chicken skin, chicken meat, fowl organ meat, powdered milk
*S*. Isangi	1	+	-	-	Chicken meat
*S*. Javiana	1	+	-	-	Wild betal leaf
*S*. Kentucky	1	+	-	-	Chicken meat
*S*. Liverpool	1	+	-	-	Chicken meat
*S*. Livingstone	1	+	-	-	Chicken carcasses
*S*. Locklease	1	+	-	-	Chicken egg
*S*. Londen	1	+	-	-	Sausage
*S*. Manhattan	1	+	-	-	Chicken meat
*S*. Mbandaka	2	+	-	-	Chicken meat, chicken egg
*S*. Meleagridis	1	+	-	-	Protein
*S*. Montevideo	1	+	-	-	Turkey meat
*S*. Muenchen	1	+	-	-	Chicken organ meat
*S*. Muenster	1	+	-	-	Turkey meat
*S*. Natal	1	+	-	-	Turkey meat
*S*. Newport	4	+	-	-	Chicken meat, turkey meat
*S*. Oranienburg	1	+	-	-	Chicken egg
*S*. Panama	1	+	-	-	Sausage
*S*. Paratyphi B var. Java	7	+	-	-	Chicken meat (n = 6), chicken caeca
*S*. Poona	1	+	-	-	Vietnamese Balm (herb)
*S*. Pullorum NCTC 10706	1	+	-	-	Reference strain
*S*. Reading	1	+	-	-	Turkey meat
*S*. Rissen	5	+	+	-	Chicken meat, swab from swine, pork, bovine sausage, mixed meats
*S*. Saintpaul	2	+	-	-	Chicken meat, turkey meat
*S*. Saintpaul WDCM00120	1	+	-	-	Reference Strain
*S*. Schwarzengrund	1	+	-	-	Protein powder
*S*. Senftenberg	1	+	-	-	Chicken egg
*S*. Stanleyville	1	+	-	-	Chicken meat
*S*. Tennessee	1	+	-	-	Chicken egg
*S*. Thompson	1	+	-	-	Duck meat
*S*. Urbana	1	+	-	-	Meat
*S*. Virchow	2	+	-	-	Mixed herbs, chicken meat
*S*. Weltevreden	6	+	-	-	Herbs (Basil, Vietnamese coriander, wild betal leaf, coriander, turmeric, mint)
***Salmonella* subspecies**	**n**	***invA***	**STM4200**	**SEN1392**	**Source of sample(s)**
*S*. *houtenae* NCTC 10056	1	+	-	-	Reference strain
*S*. *indica* NCTC 10458	1	+	-	-	Reference strain
*S*. *salamae* NCTC 9599	1	+	-	-	Reference strain
*S*. *arizonae* NCTC 7344	1	+	-	-	Reference strain
*S*. *diarizonae* NCTC 10381	1	+	-	-	Reference strain
***Salmonella* species**	**n**	***invA***	**STM4200**	**SEN1392**	**Source of sample(s)**
*S*. *bongori* NCTC 10946	1	+	-	-	Reference strain

**Table 3:** Testing to determine the absence of the *invA*, STM4200 and SEN1392 genes for 34 non-*Salmonella* reference strains by the developed multiplex qPCR method, where (+) indicates detection of the gene and (-) indicates the gene is not detected.

Non-target reference strains	*invA*	STM4200	SEN1392	Source of sample
*Aeromonas caviae*	-	-	-	RIVM ^d^
*Acinetobacter baumannii*	-	-	-	ATCC 19606 ^a^
*Arcobacter butzleri*	-	-	-	CCUG 10373 ^b^
*Bacillus cereus*	-	-	-	ATCC 9139 ^a^
*Bacillus cereus*	-	-	-	ATCC 6051 ^a^
*Brochothrix thermosphacta*	-	-	-	NCTC 10822 ^c^
*Campylobacter jejuni*	-	-	-	ATCC 33560 ^a^
*Campylobacter jejuni*	-	-	-	NCTC 11168 ^c^
*Campylobacter laridis*	-	-	-	NCTC 11352 ^c^
*Citrobacter freundii*	-	-	-	NCTC 6272 ^c^
*Citrobacter freundii*	-	-	-	NVWA control strain
*Clostridium perfringens*	-	-	-	NCTC 8449 ^c^
*Clostridium perfringens*	-	-	-	ATCC 13124 ^a^
*Clostridium bifermentans*	-	-	-	NCTC 1340 ^c^
*Cronobacter sakazakii*	-	-	-	NCTC 8155 ^c^
*Escherichia coli*	-	-	-	ATCC 25922 ^a^
*Escherichia coli*	-	-	-	NVWA control strain
*Escherichia coli*	-	-	-	NVWA control strain
*Enterobacter cloacae*	-	-	-	ATCC 23355 ^a^
*Enterococcus faecalis*	-	-	-	ATCC 29212 ^a^
*Enterococcus faecium*	-	-	-	ATCC 19434 ^a^
*Klebsiella oxytoca*	-	-	-	ATCC 49131 ^a^
*Klebsiella pneumoniae*	-	-	-	ATCC 13883 ^a^
*Lactobacillus casei*	-	-	-	ATCC 7496 ^a^
*Lactobacillus acidophilus*	-	-	-	ATCC 4357 ^a^
*Listeria monocytogenes*	-	-	-	NCTC 5348 ^c^
*Listeria monocytogenes*	-	-	-	NVWA control strain
*Listeria innocua*	-	-	-	NVWA control strain
*Morganella morganii*	-	-	-	RIVM ^d^
*Proteus mirabilis*	-	-	-	NCTC 11938 ^c^
*Pseudomonas aeruginosa*	-	-	-	NCTC 10662 ^c^
*Pseudomonas fragi*	-	-	-	NCTC 10689 ^c^
*Shigella flexneri*	-	-	-	NVWA control strain
*Shigella sonnei*	-	-	-	NVWA control strain
*Serratia marcescens*	-	-	-	ATCC 13880 ^a^
*Staphylococcus aureus*	-	-	-	ATCC 25923 ^a^
*Staphylococcus aureus*	-	-	-	NVWA control strain
*Staphylococcus epidermidis*	-	-	-	ATCC 12228 ^a^
*Vibrio parahaemolyticus*	-	-	-	ATCC 17802 ^a^
*Vibrio cholerae*	-	-	-	NCTC 11348 ^c^
*Yersinia enterocolitica*	-	-	-	CCUG 8239 A ^b^
*Yersinia enterocolitica*	-	-	-	CCUG 8233 ^b^
*Yersinia enterocolitica*	-	-	-	NVWA control strain
*Yarrowia lipolytica*	-	-	-	NVWA control strain

^a^American Type Culture Collection

^b^Culture Collection Universety Göteborg

^c^National Collection of Type Cultures

^d^National Institute for Public Health and the Environment

Strains were taken from the -80°C freezer, streaked on TSA agar, and grown aerobically for 24 ± 2 h at 37 ± 1°C. Afterwards five colonies per plate were selected, suspended in 500 μl of nuclease free demineralised water, and lysed at 95°C for 10 min using a thermomixer to obtain genomic DNA. After centrifugation at 10,000 RPM for 5 minutes, 2.5 μl of the supernatant (excessive quantity of DNA) was used as template DNA for qPCR amplification using the qPCR method developed in this study.

### LOD

The minimum level of *Salmonella* present in a (not-enriched) food product successfully detected after enrichment of the product, and subsequent DNA extraction and analysis by the qPCR method, was determined.

This level of detection (LOD) was also determined for two culture based methods, the *Salmonella* ISO 6579:2002 method [18] and the MSRV method for *Salmonella* [18], in order to compare the performance characteristics of the methods. Ten different matrices were artificially contaminated with target levels of 3, 10 and 30 CFU per volume of matrix. The matrices to be investigated were poultry meat, minced meat, egg, herbs/spices (represented by curcuma spice), powdered milk, fish (shrimps), animal feed, socks with chicken feces, swabs with debris and chicken down.

Unless otherwise stated, all media for analysis were obtained from Biotrading Benelux B.V., Mijdrecht, The Netherlands. For each matrix and level of contamination, six samples were prepared. Matrices were artificially contaminated using strain *S*. Enteritidis WDCM 00119. The strain was taken from the -80°C freezer, one bead from the cryotube was added to a test tube containing 9 ml BPW, and the tube was incubated overnight for 24 ± 2h at 37 ± 1°C. The overnight culture was serially diluted in BPW to obtain a range of concentrations. An appropriate dilution was chosen to contaminate the matrices at concentration levels of 3, 10 or 30 CFU per matrix volume. For solid and liquid matrices, 25 g or ml was contaminated. For the matrix swabs, one swab representing an area of 400 cm^2^ was contaminated. For the matrix bootsocks one pair of socks was contaminated.

For the matrix swabs and bootsocks, 225 ml of BPW was added to the artificially contaminated matrix. The other artificially contaminated samples were diluted ten times in BPW. All diluted samples were incubated for 18 ± 2 h at 37 ± 1°C. After incubation, three subsamples per inoculation level were taken from the enrichment and the presence of *Salmonella* was investigated using method ISO 6579:2002 [18], the MSRV method from ISO 6579:2002 Annex D [18] and the newly developed multiplex qPCR method. One isolate per positive sample was also tested with the qPCR method to confirm the strain was *S*. Enteritidis as added for the artificial contamination. Of every sample of the lowest detected contamination level the *Salmonella* positive isolates were also serotyped according to ISO/TR6579-3 [26]. Method ISO 6579:2002 and the MSRV method from ISO 6579:2002 Annex D were performed as described in the mentioned ISO standard. For the ISO 6579:2002 method, Rappaport-Vassiliadis Soya broth (RVS) and Muller Kauffmann TetraThionate novobiocin broth (MKTTn) were used for selective enrichment, followed by isolation of *Salmonella* on Xylose Lysine Deoxycholate (XLD) agar plates and *Salmonella* chroMID (SMID2) agar plates (Biomerieux, Zaltbommel, The Netherlands). For the MSRV method, MSRV agar plates (Oxoid, Basingstoke, England) were used for selective enrichment followed by isolation of *Salmonella* on Brilliant Green Agar (BGA) and Mannitol Lysine Crystal Violet Brilliant Green Agar (MLCB) agar plates.

In addition to direct testing of the samples after enrichment, the samples were stored cold for 48 hours (4 ± 3°C) and analyzed again afterwards to investigate the effect of cooled storage on the LOD of the detection methods.

## 3. Results and discussion

### Primer and probe analysis

BlastN analyses on a database containing 445 *Salmonella* entries retrieved 444 100% identity hits for the *invA* primers. For the *invA* probe, 442 100% hits were identified, whereas two hits were observed with one mismatch (one nucleotide difference) with the probe-sequence. Both hits with a mismatch belonged to *S*. *arizonae* isolates. For one entry of the database no hits were found with either the *invA* primers or the probe. This concerned a *S*. Senftenberg entry, which strain is known to lack *invA* in specific isolates [27].

*In silico* analyses by BlastN of the primers and probe targeting SEN1392 for *S*. Enteritidis identification on the *Salmonella* dataset identified 211 entries with 100% identity for all three oligo’s. Among these 211 were 204 Enteritidis entries, which comprises all Enteritidis entries in the dataset. Among the seven other entries with 100% hits were three *Salmonella* entries with no serotype indication and four *Salmonella* entries with a serotype other than Enteritidis (1 *S*. Moscow, 1 *S*. Nitra, 1 *S*. Blegdam, and 1 *S*. Bovismorbificans (contains one mismatch with the forward primer)). SEN1392 was described as a target region in a conventional multiplex PCR before, with similar specificity results [24].

For *S*. Typhimurium, 54 hits with 100% identity were identified with both primers and the probe targeting STM4200. This included 47 out of the 49 *S*. Typhimurium entries in the dataset. The other seven 100% hits were *Salmonella* entries without a serotype indication. Additional SeqSero analysis [28] identified the two *S*. Typhimurium genome sequences without a BlastN hit for the *S*. Typhimurium oligo’s, as not belonging to serotype *S*. Typhimurium. According to our knowledge, STM4200 was not used as a target region in a *Salmonella* PCR assay before.

No additional hits for the *invA* primers with 100% identity were obtained in a general database search. For both *S*. Enteritidis and *S*. Typhimurium, no additional entries were observed to have hits with the three oligo’s, respectively. This was except one entry, Triticum aestivum (common wheat), which was observed to have a hit with all three oligo’s for both *S*. Enteritidis and *S*. Typhimurium. However, the respective hits for the three oligo’s were not situated in proximity on the genome, inferring that no amplification product will be formed.

The *in silico* results indicate that no false negative results will be obtained by use of these oligo combinations and only a marginal number of false positive results for the *S*. Enteritidis oligo set. Therefore, the designed oligo sets appear suited for the designated use and targeting these novel genes provides innovation to this study. The designed oligo sets were further studied by performing inclusivity and exclusivity tests as reported below.

### Multiplex qPCR assay development

Analysis of the PCR products of the reference strains on the Bioanalyzer showed that each of the three primer sets produced a single DNA fragment of the expected size as listed in Table 1. The total volume of the qPCR reaction was 25 μl, containing 2.5 μl of DNA extract that was added to a mixture containing 12.5 μl 2x QuantiTect Multiplex PCR NoRox Master Mix (QIAGEN, Germany), and the respective primer and probe concentrations as listed in Table 1. The amplification conditions were an initial Taq polymerase activation for 15 min at 95°C, followed by 40 cycles for 60 s at 94°C, and a final annealing/extension step for 60 s at 60°C.

**Fig 1:**
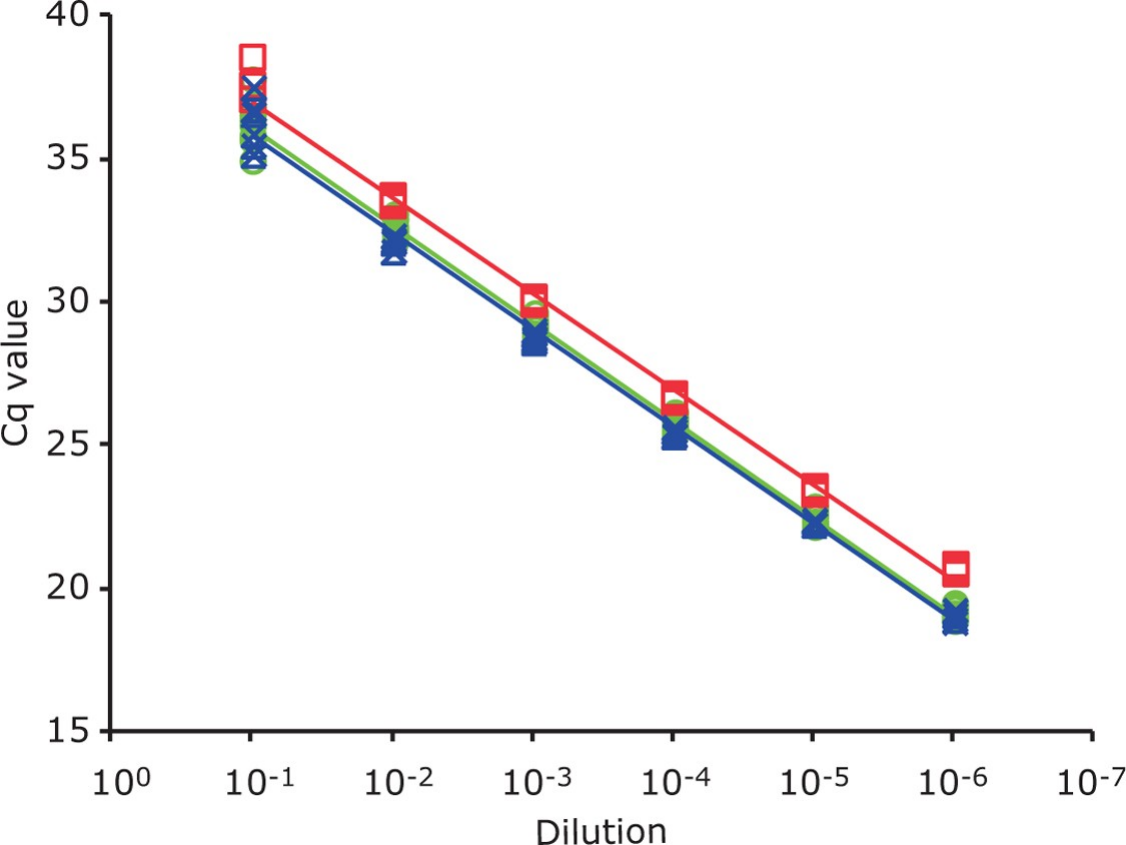
Standard curves for targets *invA*, SEN1392, and STM4200. *invA*: green line and symbols; SEN1392: red line and symbols; STM4200: blue line and symbols.

Standard curves were constructed for each target gene (Fig 1) and the slopes of the standard curves were close to the theoretical optimum of -3.32 (-3.32 for *invA*, -3.38, for SEN1392 and -3.31 for STM4200). The PCR efficiency (*E*) was calculated from the slopes using the formula *E* = (10^−1/slope^)-1 which showed that the amplification rates are very efficient, namely 100.3% for *invA*, 103.8% for SEN1392 and 99.4% for STM4200. Furthermore, the linear regression coefficient (*R*^*2*^) values of the standards curves were >0.9, indicating high linearity.

### Relative accuracy

**Fig 2:**
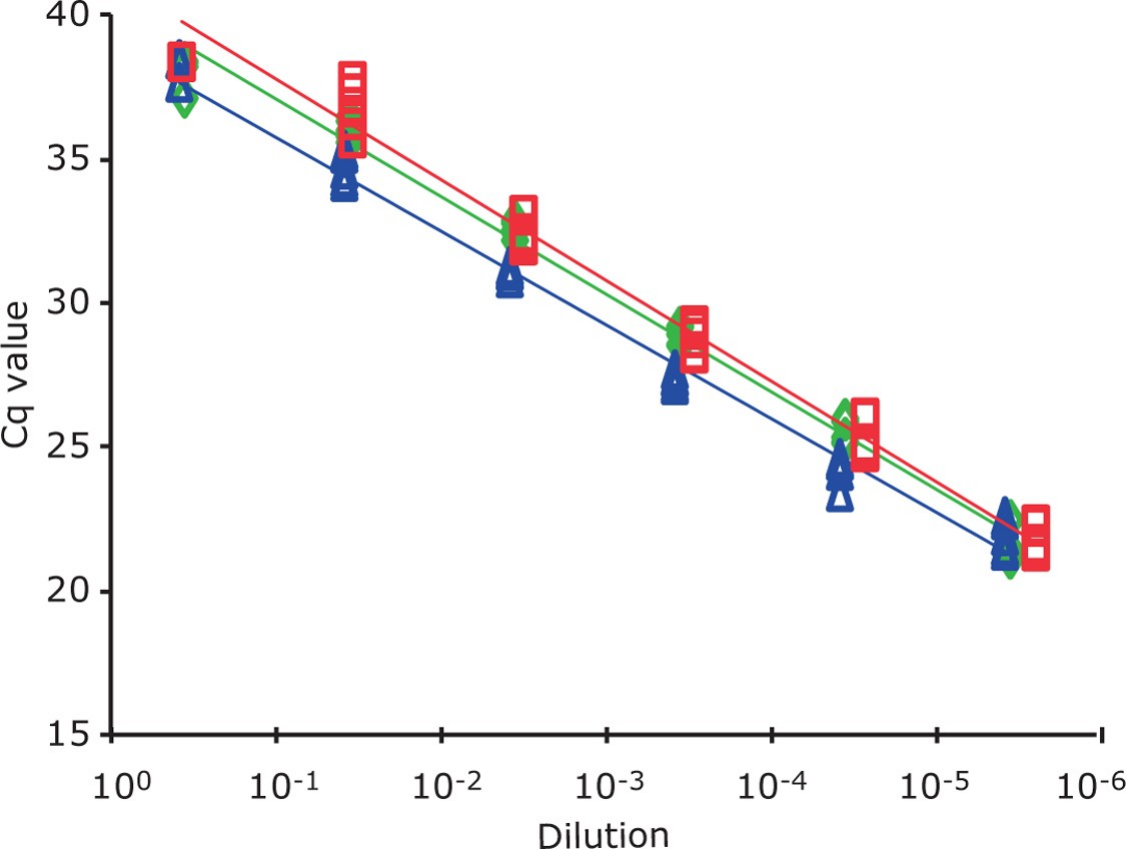
Cell standard curves for target *invA* from *S*. Typhimurium STM4200 for the matrices BPW, Chicken, and curcuma spice. BPW: Blue line and symbols; Chicken: red line and symbols; curcuma spice: green line and symbols.

To determine if there was a linear relationship between the C_q_-value and the log value of the cell numbers, a cell standard curve was constructed for *S*. Typhimurium WDCM 00031 with and without the presence of matrix. Fig 2 presents the results for the target *invA*. There was a linear relationship between cell numbers and C_q_-values both with and without matrix. The detection limit was identical for BPW and the two matrices tested and no matrix effect was observed. The limit of detection was around 3 CFU per PCR reaction, meaning that around 1200 CFU per ml enrichment need to be present in order to be detected by qPCR. Considering that enrichment takes place for 18–24 h, 1 cell present in the sample to be investigated should be able to reach this level. However, in case of severely stressed cells, cells might not exit the lag phase, or grow very slow and this should be considered when screening for *Salmonella*.

### Selectivity

The inclusivity of the *Salmonella* multiplex qPCR assay was examined by amplification of DNA isolates of different *Salmonella* serovars. All *Salmonella* strains were expected to possess the *invA* gene. In addition, *S*. Typhimurium and *S*. Enteritidis were expected to contain the STM4200 and SEN 1392 gene, respectively. The *invA* primer set was tested with a panel of 225 *Salmonella* isolates, including the different *Salmonella* (sub)species, and all strains were positively identified (Table 2). The 72 *S*. Typhimurium isolates and 53 *S*. Enteritidis isolates were positively identified by primer set STM4200 and primer set SEN1392, respectively. These results indicate 100% inclusivity for *invA*, STM4200 and SEN1392.

To determine whether the STM4200 primers could differentiate *S*. Typhimurium from the other included *S*. *enterica* serovars and (sub)species, an exclusivity panel of 94 isolates of 49 different *S*. *enterica* serovars, and an additional set of six *Salmonella* reference isolates containing the five *Salmonella* subspecies and *S*. *bongori*, were tested using the assay (Table 2). Oligo set STM4200 resulted in negative results for all six (sub)species and for 46 of the 49 different *S*. *enterica* serovars, indicating an exclusivity of 94.6%. The serovars *S*. Derby (n = 2), *S*. Goldcoast (n = 1) and *S*. Rissen (n = 5) were not excluded by the STM4200 primer set. The latter two serovars are occasionally identified in poultry meat [29,30]. As the PCR screening concerns the first phase of the *Salmonella* diagnostic algorithm for the detection of *Salmonella* in food products, and poultry products in special, an inclusivity of 100% and an exclusivity of 94.6% are acceptable and strongly indicative for the presence of *S*. Typhimurium. Nevertheless, it is highly recommended to confirm isolates that are suspected to be positive for *S*. Typhimurium by additional *Salmonella* serotyping, such as agglutination or by molecular techniques (sequencing or microarray platform), to avoid reporting false positive results. Primer set SEN1392 tested negative for all 49 *Salmonella* serovars and (sub)species included in the study.

The detection of additional *S*. *enterica* serovars by the STM4200 PCR indicates a limitation of the use of the PCR. However, the presented multiplex qPCR is clearly tested more extensively than other published *Salmonella* qPCR methods. *S*. *enterica* contains many closely related species, indicating that other qPCR methods would probably also detect additional serovars when tested as extensive as in this study.

Primer sets *invA*, STM4200, and SEN1392 were also tested with a panel of 35 non-*Salmonella* strains (Table 3). None of the primer sets tested positive with the non-*Salmonella* strains, resulting in an exclusivity of 100% for non-*Salmonella* strains.

For five proficiency tests the results were as expected. In one proficiency test one *Salmonella* strain was not detected by the qPCR method. The strain, *S*. Senftenberg, originated from cocoa and sequencing of the strain showed that the virulence-island SPI-1 (that contains the *invA* gene) was not present in this *S*. Senftenberg strain. The absence of this island in *S*. Senftenberg has been shown before [27]. Targeting *invA* in the qPCR results in a high detection rate of *Salmonella* (>99%), also in respect to other qPCR methods [31,32]. The detection rate of these molecular tools will be higher or equal to the conventional detection methods, which are based on phenotypic traits, such as motility and/or resistance characteristics. These phenotypic characteristics are not presented unanimously among *Salmonella* isolates either.

Malorny *et al*. [31] was the first to develop a qPCR for the detection of *Salmonella* spp., but this method does not allow discrimination for *S*. Enteritidis and *S*. Typhimurium. In contrast, the qPCR developed by Maurischat *et al*. [32] offers a rapid and specific qPCR, however this qPCR does not incorporate the detection of *Salmonella* spp. in general and just covers *S*. Enteritidis, Typhimurium and its monophasic variant 4,[5],12:i:−. The qPCR method developed by Silva *et al*.[33] includes detection of *Salmonella* spp. and *S*. Enteritidis, but is not able to detect *S*. Typhimurium. The qPCR presented here has selectivity comparable to the qPCR described by Park and Ricke [34], however this qPCR was only validated using a limited set of 66 *Salmonella* isolates, whereas the presented qPCR in this manuscript was validated using 225 isolates of which 72 *S*. Typhimurium isolates and 53 *S*. Enteritidis isolates.

### LOD

For official control purposes it is necessary to know which level of contamination in a sample, before enrichment, will be detected by the method. Therefore, 10 different matrices were artificially contaminated with three levels of *Salmonella* spp. and detection after enrichment was performed using the qPCR and the ISO and MSRV method. The results per matrix, contamination level and method are displayed in Table 4. For the matrices chicken, fish, swabs, powdered milk, herbs/spices (curcuma spice), egg and feed the determined LOD values were identical for the three methods tested. For the matrix boot-socks there was a difference between the ISO and MSRV method and qPCR. The LOD for the culture-based methods was below 10 CFU/ tested portion, whereas the LOD for the qPCR was around 40 CFU/ tested portion. For the matrix down it was not possible to retrieve an LOD for the qPCR method, whereas for the culture-based methods the LOD was below 10 CFU/ tested portion. The matrix down absorbed almost all the BPW used for dilution and enrichment of the samples and therefore a proper DNA extraction was not possible, indicating a negative effect of the matrix down on the DNA extraction and qPCR performance.

**Table 4:** Level of detection for various artificially contaminated matrices for the qPCR method, the ISO method and the MSRV method.

Matrix	Contamination level^a^ (CFU/25g^b^)	Real-time PCR method	ISO method	MSRV method
Chicken	37	+ (6/6)	+ (6/6)	+ (6/6)
	11	+ (6/6)	+ (6/6)	+ (6/6)
	**4**	+ (6/6)	+ (6/6)	+ (6/6)
Fish (shrimps)	37	+ (6/6)	+ (6/6)	+ (6/6)
	11	+ (6/6)	+ (6/6)	+ (6/6)
	**4**	+ (5/6)	+ (5/6)	+ (5/6)
Powdered Milk	37	+ (6/6)	+ (6/6)	+ (6/6)
	**11**	+ (6/6)	+ (6/6)	+ (6/6)
	4	- (2/6)	- (2/6)	- (2/6)
Herbs/spices (Curcuma)	37	+ (6/6)	+ (6/6)	+ (6/6)
	**11**	+ (6/6)	+ (6/6)	+ (6/6)
	3,7	- (0/6)	- (0/6)	- (0/6)
Egg	37	+ (6/6)	+ (6/6)	+ (6/6)
	**11**	+ (6/6)	+ (6/6)	+ (6/6)
	4	- (0/6)	- (0/6)	- (0/6)
Feed	37	+ (6/6)	+ (6/6)	+ (6/6)
	**11**	+ (6/6)	+ (6/6)	+ (6/6)
	4	- (0/6)	- (0/6)	- (0/6)
Down	28	- (0/6)	+ (4/6)	+ (4/6)
	**8,5**	- (0/6)	+ (3/6)	+ (3/6)
	3	- (0/6)	- (2/6)	- (2/6)
Swabs	28	+ (6/6)	+ (6/6)	+ (6/6)
	8,5	+ (6/6)	+ (6/6)	+ (6/6)
	**3**	+ (6/6)	+ (6/6)	+ (6/6)
Minced meat	37	+ (6/6)	+ (6/6)	+ (6/6)
	11	+ (6/6)	+ (6/6)	+ (6/6)
	**4**	+ (6/6)	+ (6/6)	+ (6/6)
Boot-socks with chicken feces	**43**	+ (3/6)	+ (6/6)	+ (5/6)
	13	+ (1/6)	+ (6/6)	+ (5/6)
	**4**	- (1/6)	+ (5/6)	+ (5/6)

^a^ The level of detection is marked in boldface.

^b^ For the matrix ‘swabs’ it should read CFU/swab (400 cm^2^) and for the matrix ‘socks’ it should read CFU/pair of socks.

The presented result suggest that the qPCR method can be used to screen the matrices, with the exception of the matrix down, followed by isolation of the bacterial strain using culture-based methods for the enrichments that had a positive result in the screening. This method with screening of enrichment broth allows negative samples to be identified faster in comparison to culture-based method, since enrichment followed by screening takes 24 h, whereas enrichment followed by selective enrichment using the ISO method or the MSRV method takes at least 48 h. Cooled storage of the enrichment broths prior to DNA extraction for a maximum of 48 h gave identical LOD values compared to direct testing of the enrichment. This allows laboratories to store enrichment broths during weekends or holidays, without a negative effect on the sample result. Although earlier validation studies for screening of enrichments for presence of *Salmonella* have been performed [31,32,34], the validation is this study distinguishes itself by incorporating detection for *Salmonella* spp., *S*. Enteritidis and *S*. Typhimurium in one reaction, instead of just detection of *Salmonella* spp. [31] or *S*. Enteritidis and *S*. Typhimurium [32]. As well this method is validated for ten matrices and, therefor, can be applied to a broad range of foods, feed, environmental samples and production stage samples, whereas other methods have only been validated for chicken and on a limited number of spiked samples [33].

## 4. Conclusions

*In silico* BlastN analysis indicates primers and probe sets to be highly specific for their targets, and no a-specific signals were expected based on the results. Constructed standard curves showed the qPCR was highly efficient. There was a linear relationship between cell numbers and Cq-values both with and without matrix. The inclusivity of the multiplex qPCR was 100% for all *Salmonella* isolates, including 72 *S*. Typhimurium isolates, and 53 *S*. Enteritidis isolates. The exclusivity for *Salmonella* spp., *S*. Typhimurium, and *S*. Enteritidis was 100%, 94.6%, and 100%, respectively. No positive results were reported for non-*Salmonella* isolates.

Due to the ability of the method to differentiate *S*. Typhimurium and *S*. Enteritidis, it is a robust tool to easily detect both strains as requested by regulation No 2073/2005. Since the serovars *S*. Derby, *S*. Goldcoast, and *S*. Rissen are not excluded by the STM4200 primer set, further confirmation of the isolated strains needs to be performed, identical to the confirmation for conventional culture methods.

The LOD of the multiplex qPCR method is comparable to the ISO method and MSRV method, and allows for detect of low levels (around or below 10 CFU/25g) of *Salmonella* in various (food) matrices. By using the multiplex qPCR method, instead of conventional culture methods, for screening of enrichments broths, the analysis time of samples is reduced from 48 h to 24h. This method therefore facilitates effective and faster intervention when contaminated food products are on the market.
